# LDOC1 inhibits proliferation and promotes apoptosis by repressing NF-κB activation in papillary thyroid carcinoma

**DOI:** 10.1186/s13046-015-0265-z

**Published:** 2015-12-04

**Authors:** Shuiying Zhao, Qingzhu Wang, Zhizhen Li, Xiaojun Ma, Lina Wu, Hongfei Ji, Guijun Qin

**Affiliations:** Division of Endocrinology, Department of Internal Medicine, The First Affiliated Hospital of Zhengzhou University, Zhengzhou, 450000 China; Institute of Clinical Medicine, The First Affiliated Hospital of Zhengzhou University, Zhengzhou, 450000 China

**Keywords:** LDOC1, NF-κB, Papillary thyroid carcinoma, TGF-β1

## Abstract

**Background:**

The incidence of thyroid cancer has progressively increased over the past few decades, and the most frequent types of this cancer are papillary thyroid carcinoma (PTC) and small primary tumors. In PTC, oncogene activation is known to occur at a high frequency. However, the potential roles of tumor suppressor genes in thyroid carcinogenesis remain unclear. *LDOC1* was first identified as a gene encoding a leucine zipper protein whose expression was decreased in a series of pancreatic and gastric cancer cell lines. In this study, we aimed to determine the status of LDOC1 in PTC and identify its mechanistic role in PTC pathogenesis.

**Methods:**

LDOC1 expression was evaluated in fresh samples and stored specimens of human PTC and contralateral normal tissues by performing quantitative reverse transcription-PCR and immunohistochemical staining. The correlation to nuclear p65 content in the stored specimens was analyzed. Moreover, the basal level of LDOC1 in two human PTC-derived cell lines (BCPAP and TPC-1) compared with normal thyroid tissue was determined. Human *LDOC1* cDNA was inserted into a lentiviral vector and transduced into TPC-1 cells. TPC-1 cells overexpressing LDOC1/GFP (Lv-LDOC1) or negative control GFP (Lv-NC) were stimulated with TNFα or recombinant TGF-β1, and then cell proliferation, cell cycle distribution, and apoptosis were assessed. Western blotting was used to examine the expression of p65, IκBα, c-Myc, Bax, and Bcl-xL, and a luciferase reporter assay was used to measure NF-κB activity stimulated by TNFα. Statistical significance was determined using Student’s *t* tests or ANOVA and Newman-Keuls multiple comparison tests. Pearson chi-square test was used to analyze possible associations.

**Results:**

LDOC1 expression was significantly downregulated in PTC specimens as compared with the expression in normal thyroid tissues, and this downregulation was associated with an increase in tumor size (*P* < 0.05). There is a correlation between LDOC1 and nuclear P65 expression in human PTC tissues (*P* < 0.01). Lentivirus-mediated restoration of LDOC1 expression in TPC-1 cells characterized by low level of LDOC1 expression suppressed proliferation and induced apoptosis by inhibiting NF-κB activation, and LDOC1-overexpressing TPC-1 cells recovered responsiveness to TGF-β1 antiproliferative signaling.

**Conclusions:**

LDOC1 might function as a tumor suppressor gene in PTC by inhibiting NF-κΒ signaling, and thus might represent a promising therapeutic target in patients with PTC.

## Background

Thyroid cancer is currently the most common endocrine malignancy, and its incidence accounts for >5 % of all cancers in women [1]. Papillary thyroid carcinoma (PTC), the most prevalent histotype among thyroid malignancies [2], is typically well-differentiated and associated with a favorable therapeutic response and prognosis; however, in the case of aggressive PTC and certain PTC variants, around 10 % of the patients manifest recurrences or distant metastases within 10 years [3–5]. Thus, although the roles of several specific genes in PTC have been established, other biomarkers are required to improve patient stratification for the purpose of determining the most effective therapeutic options and follow-up strategies and identifying novel therapeutic targets for PTC.

Various oncogenes have been identified to be involved in human thyroid cancers, particularly in the papillary histotype [6, 7]. Mutations in the gene *BRAF* represent the predominant molecular alterations in PTCs [8, 9], and activation of the *RET/PTC* oncogene [10] has been identified in approximately 20 % of PTCs. In tumor progression in general, the early stages are characterized by both the silencing of tumor suppressor genes [11, 12] and an enhanced expression of oncogenes [13–15]. In the case of PTC in particular, oncogene activation is known to occur at a high frequency, but the tumor suppressor genes involved remain poorly elucidated.

Leucine zipper downregulated in cancer 1 (*LDOC1*) was originally identified using differential RNA display to be downregulated in a series of pancreatic and gastric cancer cell lines [16]. The LDOC1 protein contains a DNA-binding leucine zipper-like motif and a short proline-rich region. LDOC1 downregulation and differential expression have also been characterized in tissue samples of esophageal cancer [17] and various types of leukemia [18], which suggests that LDOC1 likely functions as a tumor suppressor. Furthermore, LDOC1 has been described to regulate NF-κB activation in human cancer cells [19, 20] and biliary epithelial cells [21]. The NF-κB signaling pathway is widely recognized to play a major role in the initiation and progression of thyroid carcinoma [22–26]. However, the expression and function of LDOC1 in PTC remain unclear.

In this study, we validated the downregulation of LDOC1 in human PTC tissue samples and its relationship to tumor size and nuclear p65 content. We further show that LDOC1 can suppress PTC cell growth and induce apoptosis. Notably, LDOC1 sensitizes PTC cells to apoptosis by regulating NF-κB activation, and, consequently, modulates the responsiveness of these cells to TGF-β1 antiproliferative signaling. These data suggest that LDOC1 functions as a putative tumor suppressor gene in thyroid tumorigenesis.

## Methods

### Thyroid tissue specimens

Human thyroid tissue samples were collected at The First Affiliated Hospital of Zhengzhou University between 2013 and 2014. Fresh PTC tissue specimens (*n* = 126) and control samples (contralateral normal thyroid lobe, *n* = 47) were frozen in liquid nitrogen immediately after thyroidectomy and stored at −80 °C. These samples were analyzed using quantitative reverse transcription-PCR (qRT-PCR). Stored, formalin-fixed, paraffin-embedded cancer tissues obtained from 56 patients with PTC were investigated using immunohistochemical staining, with 33 contralateral normal thyroid specimens serving as control samples. All tissue samples used in this study were diagnosed by two endocrine pathologists, and representative tumor areas were selected for analysis.

This study was conducted in accordance with the Helsinki Declaration and was approved by the Ethics Committee of The First Affiliated Hospital of Zhengzhou University. All participants provided written informed consent for the use of the samples in the study.

### Thyroid cell line and cell culture

The PTC-derived thyroid cell line TPC-1 was kindly provided by Professor Li-li Zheng (The First Affiliated Hospital of Zhengzhou University, Zhengzhou, China). Cell line BCPAP was obtained from Chinese Academy of Sciences (Shanghai, China). Cells were cultivated in DMEM supplemented with 10 % fetal calf serum in a 5 % CO2 atmosphere at 37 °C.

### Lentivirus-mediated LDOC1 overexpression in TPC-1 cells

Human *LDOC1* cDNA was amplified, purified, and inserted into a lentiviral vector encoding enhanced GFP [27, 28]. The recombinant lentiviral vector expressing LDOC1 (Lv-LDOC1) and the empty vector (Lv-NC; negative control, encoding GFP alone) were constructed by Shanghai GenePharma Co., Ltd. (Shanghai, China) and verified by sequencing. Packaging, purification, and titer determination of the recombinant lentiviruses were implemented in HEK293T cells as previously described [29, 30]. In two precise preparations, the titers of the recombinant lentiviruses were 2 × 10^8^ and 5 × 10^8^ infectious units/mL, respectively.

TPC-1 cells were cultured in 24-well plates and infected with the lentivirus at a multiplicity of infection (MOI) of 20, 50, or 100 for 24 h. Flow cytometry analysis revealed that approximately 95 % of the cells were infected at an MOI of 50. To obtain stable LDOC1 expression, cells were selected using puromycin (Sigma-Aldrich, St. Louis, MO, USA), and LDOC1 expression in the infected cells was validated by means of qRT-PCR and western blotting.

### RNA isolation and qRT-PCR

Total RNA was extracted from frozen samples of human tissue and from cells by using TRIzol reagent (Invitrogen, San Francisco, CA, USA) according to the manufacturer’s instructions. RNA integrity was assessed by performing denaturing agarose-gel electrophoresis, and then 1 μg of total RNA was used for first-strand cDNA synthesis by using the Superscript III First-Strand Synthesis system (Toyobo, Osaka, Shiga, Japan); the volume of each reaction was 10 μL, and cDNA synthesis was performed under these thermal conditions: 37 °C for 15 min, 50 °C for 5 min, and 98 °C for 5 min. qPCR was performed using Fast SYBR Green Master Mix, as per manufacturer instructions (Applied Biosystems Inc., Foster City, CA, USA), and β-actin was used for normalization. The amplification conditions were the following: 1 min at 95 °C, followed by 40 cycles of 15 s at 95 °C and 1 min at 60 °C.

The *LDOC1* primers were (forward) 5′-CTA TGC TGC CAC TTC ACA TCC-3′ and (reverse) 5′-GTG AGC TGT CCA AAT CAA TGT C-3′. Each reaction was performed in triplicate. We used the 2^-Δ CT^ method to calculate the relative expression levels of the target gene. *LDOC1* expression in thyroid tissues was log_2_-transformed and expressed as the *LDOC1*/β-actin expression ratio (−Δ CT).

### Antibodies, western blot analysis, and immunohistochemistry

Primary antibodies against the following proteins were from commercial sources: p65 (F-6; sc-8008), IκBα (6A920; sc-56710), tubulin (sc-5286), and Bcl-xL (sc-1041), Santa Cruz Biotechnology (Dallas, TX, USA); β-actin (D110024) and Bax (AB20073b), Sangon Biotech (Shanghai, China); cleaved PARP (Asp214; #9541) and cleaved caspase-3 (Asp175, 5A1E; #9664), Cell Signaling Technology (Beverly, MA, USA); and LDOC1 (ab98390), Abcam (Cambridge, UK). Goat anti-rabbit IgG-horseradish peroxidase (HRP) (sc-2030) and goat anti-mouse IgG-HRP (sc-2031) secondary antibodies were from Santa Cruz Biotechnology.

Cytoplasmic and nuclear protein extracts were obtained using a ProteoJET Cytoplasmic and Nuclear Protein Extraction Kit (Fermentas, Glen Burnie, MD, USA), according to the manufacturer’s instructions. Total protein (30–50 mg) or proteins from the nuclear/cytoplasmic fraction (15 mg) were separated by means of SDS-PAGE and then transferred onto PVDF membranes. The membranes were incubated with primary antibodies overnight at 4 °C, after which the target proteins were visualized by incubating the blots with species-specific HRP-conjugated secondary antibodies and then with an enhanced chemiluminescence reagent (Amersham Biosciences Corp., Piscataway, NJ, USA). Antibodies against β-actin, tubulin, and histone H2A were used as controls to normalize protein input. Western blot bands were quantified using Image J analysis software.

We stained 5-μm-thick thyroid tissue sections by using the ABC reagent kit (Vector Laboratories, Burlingame, CA, USA) as specified by the supplier. LDOC1 expression in normal and malignant samples was semi-quantitatively scored according to methods presented by Pinheiro et al. [31]. The final degree of immunoreactivity was determined by the sum of the staining intensity and the area of positive staining; samples were grouped according to negative (0), weak (1–2), moderate (3), or strong (4–6) staining. The positive-staining category included only samples that exhibited moderate and strong staining; samples that received other final scores were regarded as negative. For p65 expression, only nuclear immunoreactivity was regarded as constitutive NF-κB activation and specimens were considered positive if 10 % or more of tumor cells were nuclear stained as previously described [32].

### Cell cycle analysis

To monitor LDOC1-induced changes in the cell cycle, 2 × 10^5^ cells were plated per well in 6-well plates overnight and then serum-starved for synchronization in G0/G1 phase. After synchronization for 24 h, fetal bovine serum (FBS) was added to the cultures to promote cell cycle reentry and the cells were concomitantly treated with recombinant human TGF-β1 (rTGF-β1; Peprotech, Rocky Hill, NJ, USA). Subsequently, the cells were fixed at various times and stained with propidium iodide (BB-4104-2; BestBio, Shanghai, China) according to the manufacturer’s procedures, and then measured using flow cytometry analysis performed on a Guava EasyCyte Mini System (Guava Technologies, Billerica, MA, USA).

### Cell proliferation assay

Cell viability and cell numbers were assessed using Cell Counting Kit-8 (CCK-8) (BB-4202-2; BestBio, Shanghai, China); assays were performed in 96-well plates in triplicate. Cells were seeded at 1000 cells/well in 200 μL of culture medium the day before treatment. Cell numbers were determined daily for 7 days after seeding, or the cells were treated with a stimulator (TNFα or rTGF-β1) at various times and then the 450-nm absorbance of the samples was measured using a 96-well microplate reader.

### Apoptosis assay

TPC-1 cells (2 × 10^5^ cells/well) infected with Lv-LDOC1 or Lv-NC were seeded in 6-well plates and serum-starved for 1 day, and then the culture medium was replaced with culture medium containing 10 % FBS; 24 h after medium replacement, apoptosis was examined using an APC-conjugated Annexin V and 7-AAD apoptosis-detection kit (BD Biosciences, Franklin Lakes, NJ, USA), according to manufacturer instructions.

### NF-κB-luciferase reporter assay

Transduced TPC-1 cells were transiently transfected with the NFκB-driven firefly-luciferase reporter vector by using the FuGENE 6 transfection reagent (Roche Diagnostics GmbH, Mannheim, Germany) according to manufacturer instructions. At 24 h post-transfection, the culture medium was replaced with serum-free medium, and 24 h later, the cells were treated with or without TNFα. The cells were then collected and analyzed for *firefly* luciferase activities by using the ONE-Glo luciferase assay system (Promega, Madison, WI, USA) according to the product manual. Transfections were performed in triplicate.

### Statistical analysis

The results are presented as means ± SEM of three independent experiments performed in at least triplicate. The data were analyzed using SPSS 19.0 software (SPSS, Chicago, IL, USA). A two-tailed Student’s *t* test was used for comparisons between two groups, and ANOVA and Newman-Keuls tests were used for multiple comparisons. Pearson chi-square test was performed to analyze possible associations between LDOC1 and nuclear p65 content in tissue samples. Graphs were generated using GraphPad Prism Software version 5.0 (San Diego, CA, USA). *P* < 0.05 was considered statistically significant.

## Results

### LDOC1 expression is reduced and is associated with both primary tumor size and nuclear p65 content in human PTC

**Fig. 1:**
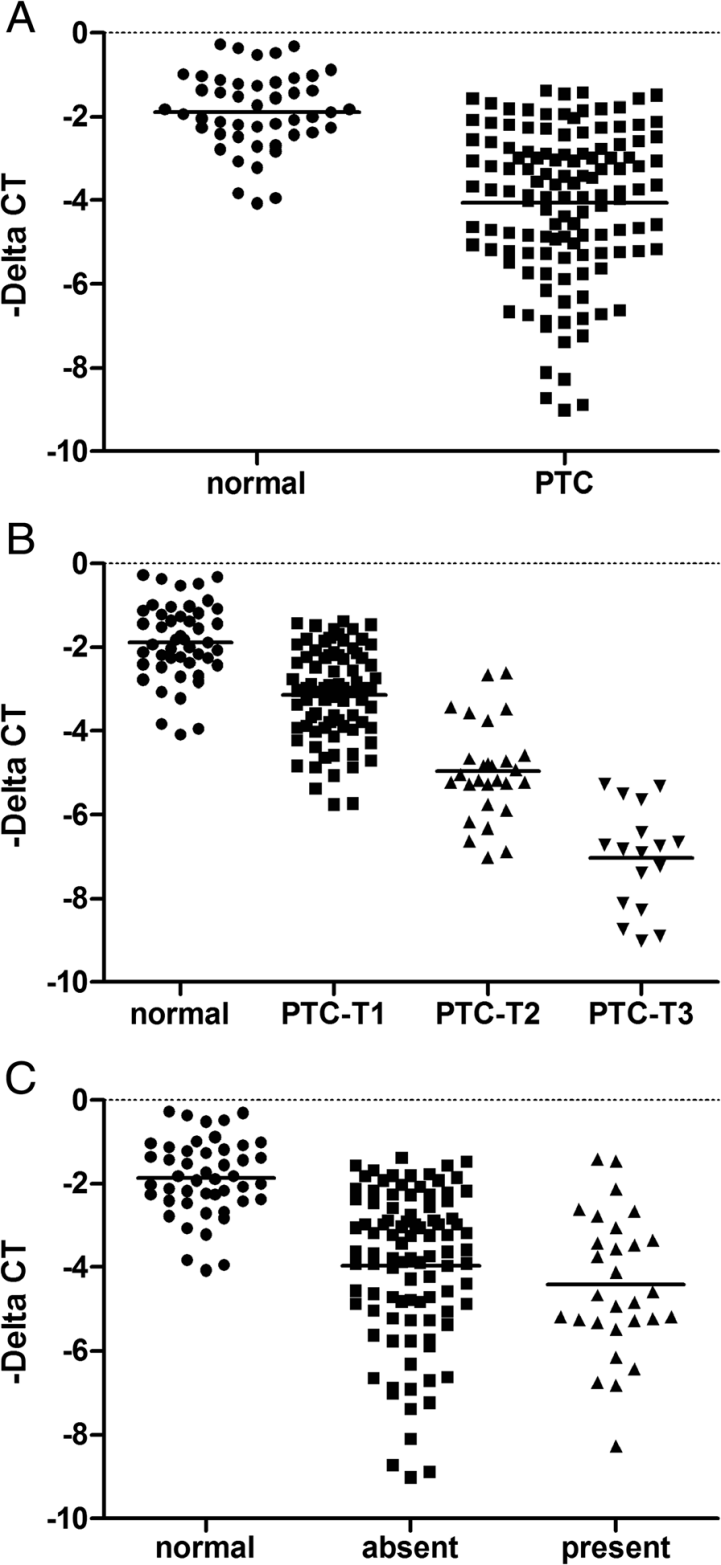
*LDOC1* mRNA expression is downregulated in papillary thyroid carcinoma (PTC) tissues. **a** *LDOC1* mRNA expression compared between PTC (*n* = 126) and normal (*n* = 47) tissue samples. **b** Comparison of *LDOC1* mRNA expression in normal and PTC samples according to tumor T status. **c** Relative expression of *LDOC1* with or without the presence of extrathyroidal extension. All quantitative reverse transcription-PCR (qRT-PCR) data are shown as means ± SEM from triplicate assays after normalization against β-actin. *LDOC1* expression is log_2_-transformed and expressed as the *LDOC1*/β-actin expression ratio (−Δ CT)

To define the expression profile of LDOC1 in human PTC tissues, qRT-PCR was performed on 126 PTC surgical specimens (Table 1) and 47 normal human thyroid tissues. *LDOC1* expression was drastically downregulated in PTC samples as compared with the expression in normal thyroids (Fig. 1a), but *LDOC1* expression did not differ markedly between classic PTCs and other histological PTC variants (data not shown). Decreased *LDOC1* mRNA expression correlated inversely with increased primary tumor size in PTC (Fig. 1b); however, low *LDOC1* expression was not associated with the presence of extrathyroidal invasion (Fig. 1c).

**Table 1:** Clinicopathological features of the study cohort

Characteristics	Number
No. of patients	126
Age (mean ± SD), years	44.8 ± 12.7
Sex (male/female)	34/92
Histological variant (cPTC/other)	101/20
Tumor size (T1/T2/T3)^a^	81/28/17
Extrathyroidal extension (absent/present)	97/29
Distant metastasis	0

^a^Tumor size: T1 < 2 cm; T2 > 2 cm, but ≦4 cm; T3 > 4 cm

**Fig. 2:**
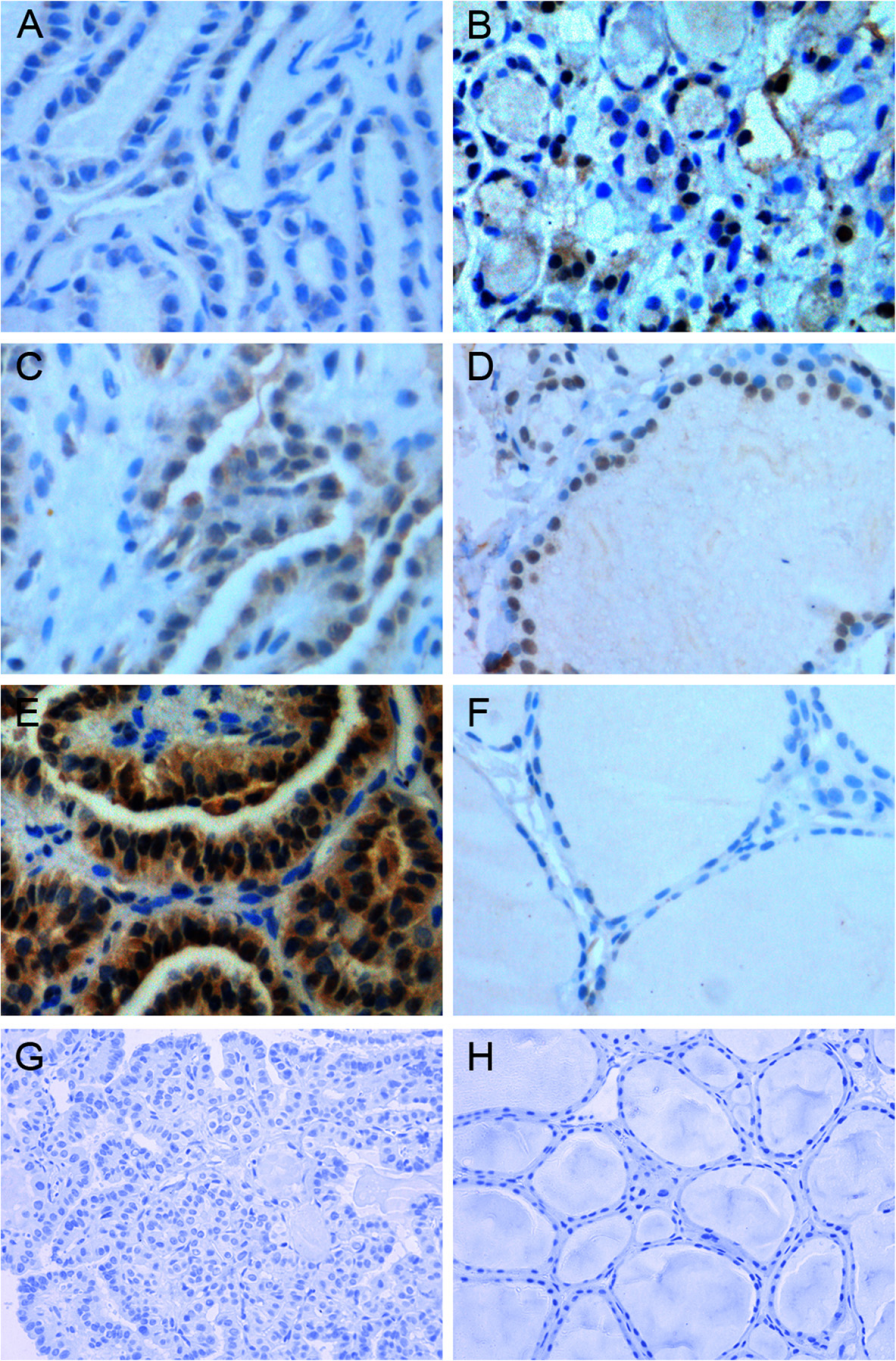
Immunohistochemical analysis of LDOC1 and P65 expression in PTC and normal samples. **a**, **c** Representative images of LDOC1 in classic PTC (400×): two different specimens. **b** Representative images of LDOC1 in follicular variants of PTC (400×). **d** Representative images of LDOC1 in normal thyroid (400×). **e** Thyroid papillary carcinoma (400x), showing nuclear P65 immunoreactivity. **f** Normal thyroid (400x), negative for nuclear P65 expression. **g**, **h** Negative controls for PTC and normal tissues (200×): staining without anti-LDOC1 antibody

Next, we analyzed LDOC1 and nuclear P65 expression levels in paraffin-embedded samples by performing immunohistochemical staining (Tables 2, 3 and Fig. 2). As expected based on the aforementioned results, LDOC1 expression was extremely low or absent in PTC samples (Fig. 2a, b) and moderate or high in normal thyroids (Fig. 2d). Notably, when LDOC1 was detectable in PTC samples, the protein was mislocalized from the nucleus to the cytoplasm (Fig. 2c). Nuclear P65 expression was observed in a high percentage of PTC (42/56, 75.0 %), whereas only 6 out of 33 cases (18.2 %) showed positive nuclear staining in normal thyroid. The specificity of the staining was validated by a lack of tissue immunoreactivity upon omission of the primary antibody (Fig. 2g, h). As shown in Table 3, the results also revealed that there is a correlation between LDOC1 and nuclear P65 expression in human PTC tissues.

**Table 2:** Immunohistochemical analysis of LDOC1 expression in human PTC

Group	No. of patients	Negative (0)	Weakly stained (1–2)	Moderately stained (3)	Strongly stained (4–6)
Cancer	56	29	18	7	2
Normal	33	1	4	13	15

**Table 3:** Correlations between LDOC1 and nuclear P65 expression in tumor tissues

LDOC1	Nuclear P65		Total	Positive rate (%)
	Positive	Negative		
Positive	3	6	9	33.3
Negative	39	8	47	83.0
Total	42	14	56	75.0

*χ* ^*2*^ = 7.458

*P* = 0.006

### Lentivirus-mediated LDOC1 stable expression in the PTC cell line TPC-1

**Fig. 3:**
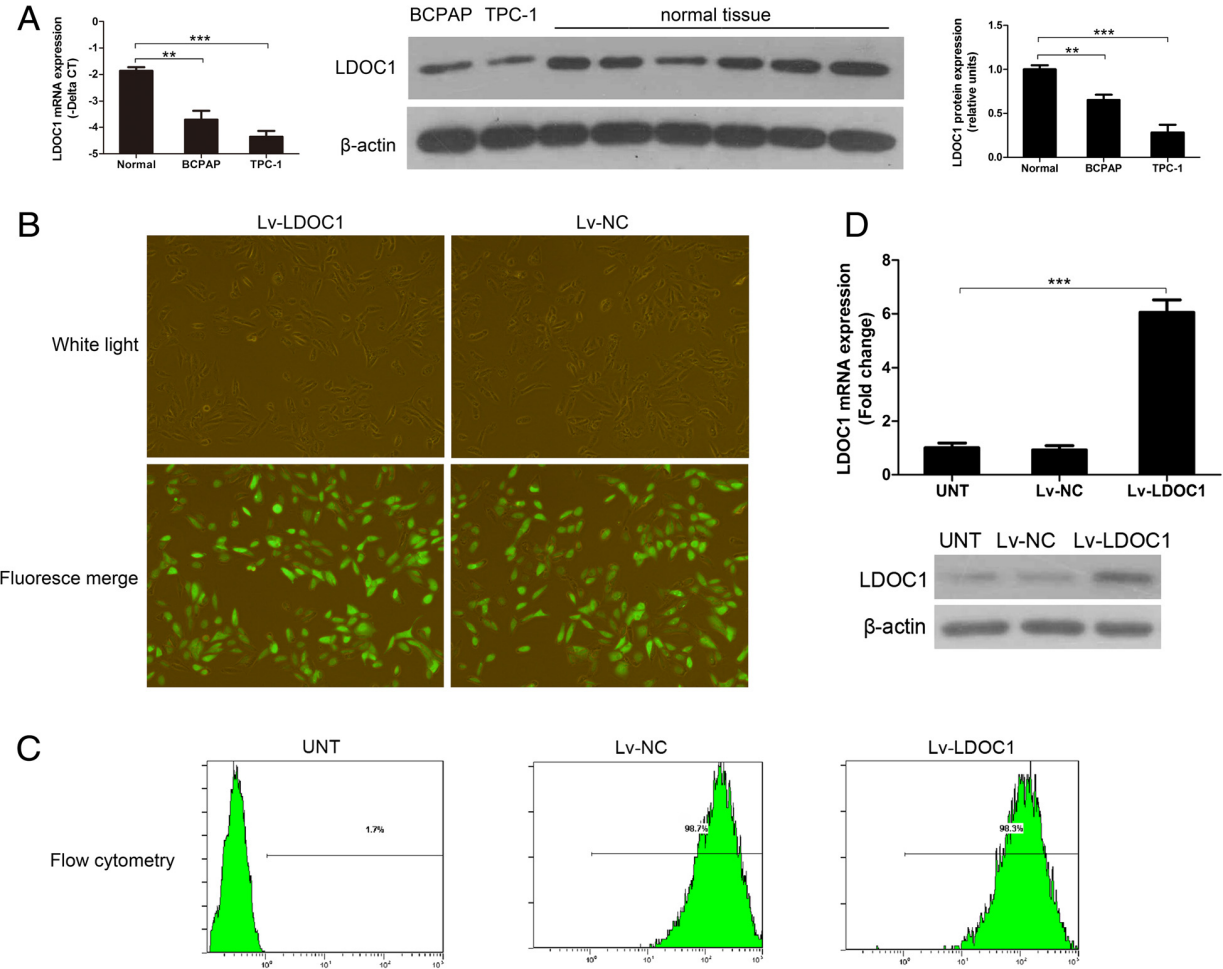
LDOC1 overexpression in the PTC-derived cell line TPC-1 by means of lentivirus-mediated cDNA expression. **a** LDOC1 expression in PTC cell lines (BCPAP and TPC-1) and normal thyroid tissue (*n* = 47). Some representative results of immunoblotting are shown. **b** Representative images of TPC-1 cells transduced with either a lentiviral vector carrying the *LDOC1* cDNA (Lv-LDOC1) or the empty lentiviral vector (Lv-NC; negative control, GFP only). **c** Flow cytometry analysis of the efficiency of lentivirus-mediated cDNA transduction. **d** LDOC1 overexpression in TPC-1 cells validated using qRT-PCR and western blotting. LDOC1 expression was normalized relative to the level of the loading control β-actin. **P < 0.01 and ***P < 0.001

We evaluated LDOC1 mRNA expression in two human PTC-derived cell lines (BCPAP and TPC-1) and there was a significantly lower expression of LDOC1 with respect to normal thyroid tissue (Fig. 3a). As a first step in determining the biological relevance of reduced LDOC1 expression in PTC, we stably overexpressed LDOC1 in TPC-1 cells, which is an authentic thyroid cancer cell line, as demonstrated by Schweppe et al. [33]. The cultured TPC-1 cells were transduced with either a lentiviral vector designed for LDOC1 stable overexpression (Lv-LDOC1) or the empty lentiviral vector (negative control, Lv-NC) (Fig. 3b), and then selection was performed using puromycin in order to enrich lentivirus-infected cells. The efficacy of lentivirus-mediated LDOC1 overexpression was validated by means of flow cytometry, qRT-PCR, and immunoblotting (Fig. 3c, d).

### Elevated LDOC1 expression suppresses proliferation and promotes apoptosis in TPC-1 cells

**Fig. 4:**
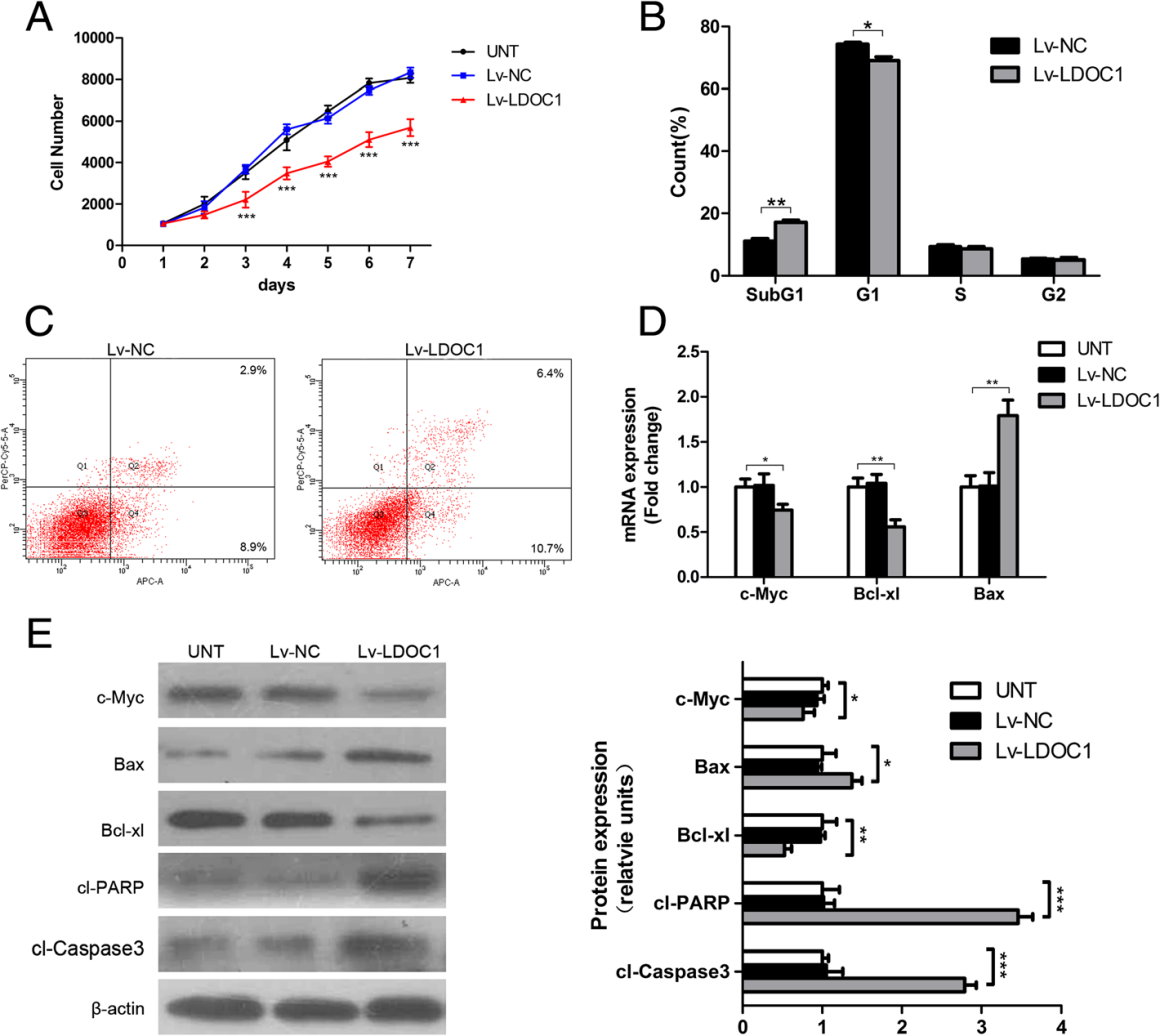
Influence of LDOC1 overexpression on TPC-1 cell growth and apoptosis. **a** TPC-1 cell numbers: UNT cells and cells transduced with Lv-LDOC1 or Lv-NC; cell numbers at the indicated times were estimated using CCK-8 proliferation assays. Compared with UNT cells, TPC-1 cells transduced with Lv-LDOC1 showed a significant reduction in cell number starting from Day 3. **b** LDOC1 restoration induces an increment of cells in subG1 phase. TPC-1 cells transduced with Lv-NC or Lv-LDOC1 were seeded into 6-well plates and cultured overnight and then serum-starved for 24 h; next, the culture medium was replaced with medium containing FBS, and after culturing for another 24 h, the cells were trypsinized and cell cycle distribution was analyzed by performing propidium iodide staining and flow cytometry. **c** Apoptosis assessment in TPC-1 cells. Cells transduced as in (**b**) were collected and stained using an Annexin V-APC/7-AAD apoptosis-detection kit. The upper and lower right quadrants display the percentages of apoptotic cells. **d** Levels of Bax, Bcl-2, and Bcl-xL determined using qRT-PCR. **e** Western blot analyses of Bax, Bcl-2, Bcl-xL, caspase-3, and PARP; β-actin was used as a loading control. *P < 0.05, **P < 0.01, and ***P < 0.001

To investigate the biological effects of LDOC1 in PTC cells, we measured the growth rate of TPC-1 cells by performing the CCK-8 assay after restoration of LDOC1 expression. Cells transduced with the *LDOC1* cDNA-harboring vector grew considerably more slowly than did untransduced (UNT) cells and cells transduced with the negative control vector (Lv-NC) (Fig. 4a); LDOC1 overexpression led to a significant reduction in TPC-1 cell number by Day 7 (*P* < 0.05). Next, we examined the molecular mechanism underlying this altered cell growth by investigating how LDOC1 expression affected the cell cycle distribution of TPC-1 cells after synchronization induced through serum deprivation. Flow cytometry analysis revealed that LDOC1 overexpression corresponded with a statistically significant shift within the DNA profile to a subG1 position; the proportion shifted from 11.8 % in the case of negative-control cells to 17.1 % for LDOC1-overexpressing cells (Fig. 4b).

We also analyzed whether LDOC1 plays a role in regulating apoptosis in TPC-1 cells. We used an Annexin V-APC/7-AAD kit for these assays, and our results revealed that the apoptotic index of Lv-LDOC1-infected cells was markedly higher than that of negative-control cells (Fig. 4c). Furthermore, we measured the levels of apoptosis-related proteins (Fig. 4d, e), and the results demonstrated that LDOC1-overexpressing TPC-1 cells expressed higher levels of cleaved caspase-3, cleaved PARP, and Bax as compared with UNT and Lv-NC cells. By contrast, Bcl-xL and c-Myc expression levels were markedly decreased (relative to control) after LDOC1 overexpression. Collectively, these results suggest that LDOC1 promotes apoptosis in TPC-1 cells.

### LDOC1 inhibits NF-κB signaling in TPC-1 cells

**Fig. 5:**
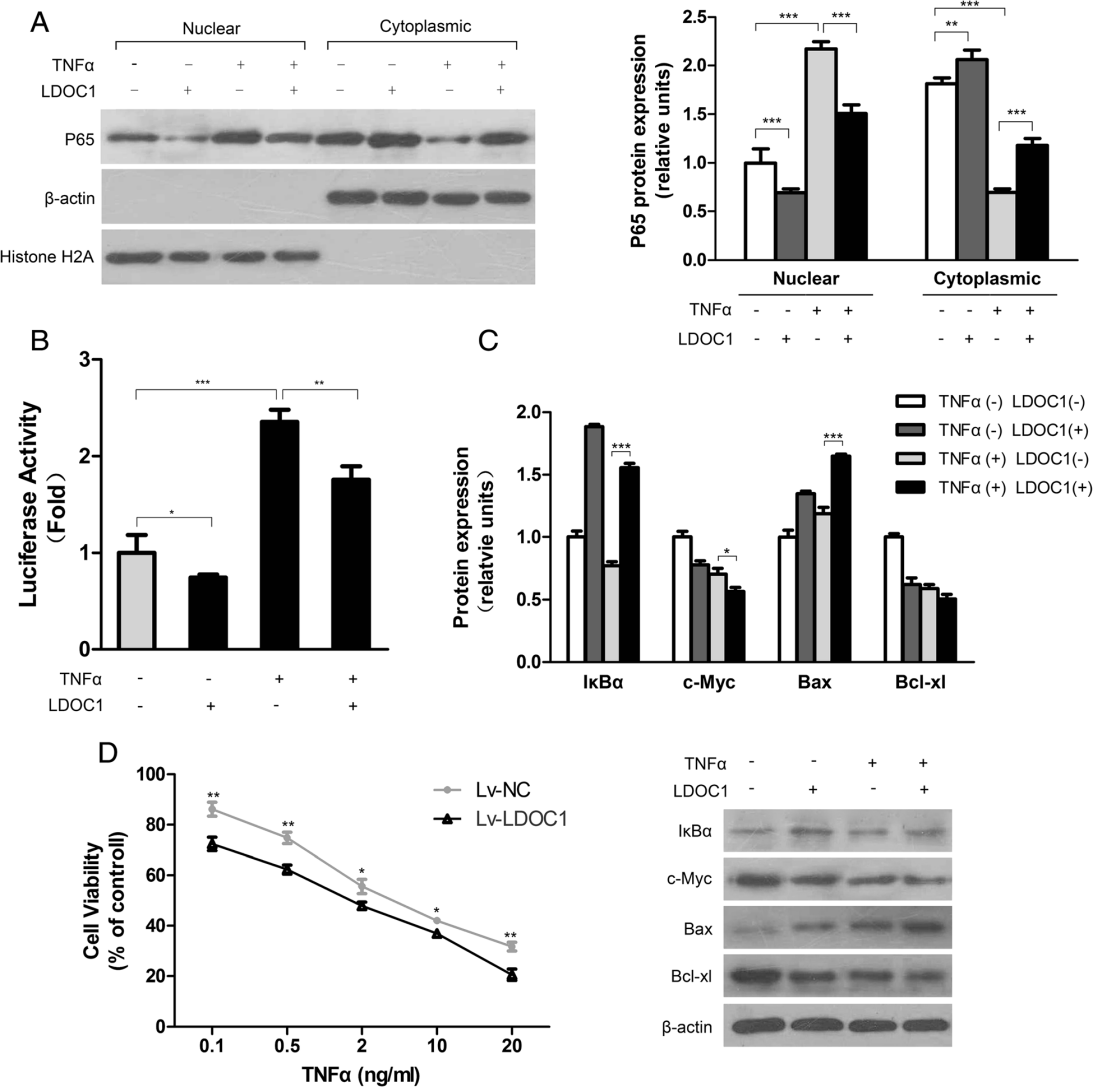
LDOC1 inhibits NF-κB activation in TPC-1 cells. **a** Immunoblotting analysis of p65 expression in the nucleus and the cytoplasm. TPC-1 cells transduced with Lv-LDOC1 or Lv-NC were stimulated with TNFα (10 ng/mL) for 30 min. Proteins from nuclear and cytoplasmic fractions were extracted and immunoblotted; histone H2A and β-actin were used as loading controls. **b** Stable LDOC1 overexpression inhibited basic and TNFα-stimulated NF-κB activation. Cells transduced as in (**a**) were transiently transfected with an NF-κB-luciferase expression vector. At 24 h after transfection, cells were serum-starved for a further 24 h, after which they were stimulated with TNFα (10 ng/mL) for 24 h and used in luciferase activity assays. Results are presented as luciferase activity values relative to the value in cells transfected with the control vector. **c** Western blot analysis of IκBα, c-Myc, Bax, and Bcl-xL in Lv-NC and Lv-LDOC1 cells. Cells were treated with TNFα (10 ng/mL) for 30 min or 24 h (IκBα), and tubulin was used for normalization. All data (**a**-**c**) are representative of three independent experiments. **d** Viability of Lv-LDOC1 and Lv-NC cells was determined using a CCK-8 proliferation assay. Cells were stimulated with the indicated concentrations of TNFα once every 2 days together with fresh medium for 5 days. The data shown represent three independent experiments performed in triplicate

Our aforementioned results imply that a loss of LDOC1 expression contributes to cell survival and protects against apoptosis. In cancer cells, one of the main signaling pathways related to survival, chemoresistance, and radiation-resistance is the NF-κB pathway. Therefore, we investigated whether LDOC1 is involved in regulating the NF-κB signal transduction pathway in PTC cells. Because NF-κB signaling in several cell types is known to be potently induced by TNFα, we tested how the restoration of LDOC1 expression affects TNFα-induced NF-κB activation in TPC-1 cells. Toward this end, we performed western blotting to examine the expression of p65, a subunit of the NF-κB transcription complex, in nuclear extracts after LDOC1 expression was restored in TPC-1 cells. LDOC1 restoration resulted in diminished nuclear NF-κB expression but elevated cytoplasmic NF-κB expression (Fig. 5a). Moreover, TNFα treatment enhanced NF-κB activation almost 2.5-fold over the basal level in Lv-NC cells, whereas this activation was significantly impaired in TPC-1 cells following lentivirus-mediated LDOC1 overexpression (*P* < 0.01), as shown by the results of an NF-κB-luciferase reporter assay (Fig. 5b). Lastly, LDOC1 also inhibited the degradation of IκBα, an NF-κB inhibitor (Fig. 5c). These results suggested that both constitutive and TNFα-inducible activation of NF-κB signaling were modulated by LDOC1 overexpression in thyroid cancer TPC-1 cells.

TNFα functions as a “double agent” that can induce both the NF-κB-dependent cell-survival pathway and the caspase-dependent apoptotic pathway [34, 35]. Therefore, we further analyzed how LDOC1 expression affected TNFα-induced antiproliferative activity in TPC-1 cells. In line with our other LDOC1 results, TNFα-induced loss of cell viability was higher in LDOC1-overexpressing TPC-1 cells than in Lv-NC cells (Fig. 5d), and the protein levels of c-Myc and Bcl-xL were also decreased significantly in the LDOC1-expressing cells (Fig. 5c). Taken together, these data suggest that LDOC1-mediated NF-κB inhibition markedly sensitized the TPC-1 thyroid cancer cells to apoptosis in response to extracellular stimulation.

### LDOC1 increases TPC-1 cell sensitivity to TGF-β1 inhibitory signaling

**Fig. 6:**
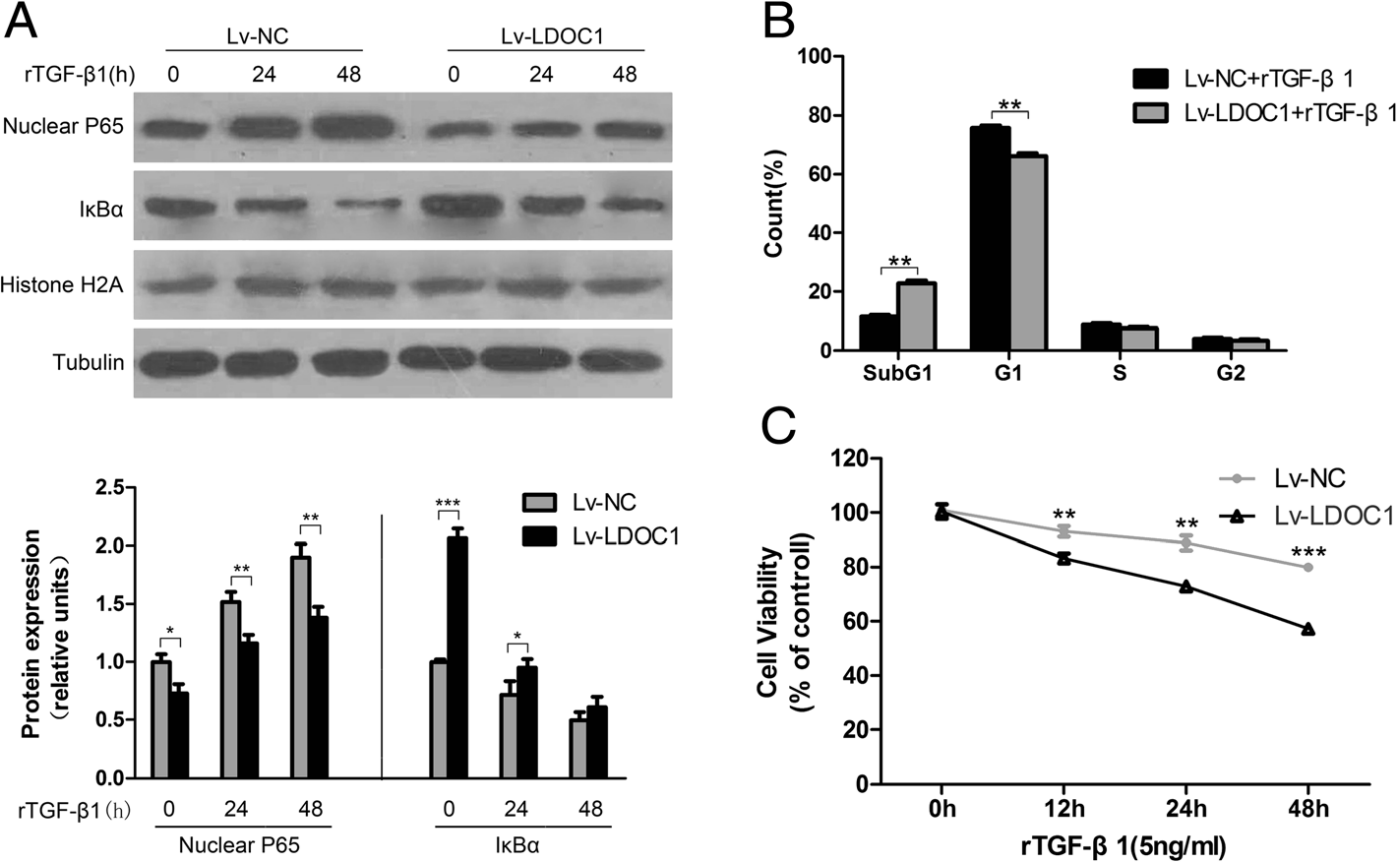
LDOC1 restores the responsiveness of TPC-1 cells to TGF-β1 inhibitory signaling. **a** TPC-1 cells transduced with Lv-NC or Lv-LDOC1 were treated with or without rTGF-β1 (5 ng/mL) for the indicated times. The levels of nuclear p65 and IκBα were determined by performing western blot analysis, with histone H2A and β-actin being used as loading controls, respectively. **b**, **c** Cell cycle arrest and cell viability were assessed in Lv-NC or Lv-LDOC1 cells treated as in (**a**). *P < 0.05, **P < 0.01, and ***P < 0.001 compared with Lv-NC cells (Student’s *t* test). All data are presented as means ± SEM from three independent experiments

TGF-β1 potently inhibits the proliferation of normal epithelial cells; however, in neoplastic thyrocytes, this antiproliferative effect of TGF-β1 can be overridden by the activation of the NF-κB survival pathway [36]. Because the NF-κB pathway mediates the effect of TGF-β1 on survival, we investigated whether LDOC1 enhances the sensitivity of TPC-1 cells to TGF-β1 inhibitory signaling by suppressing NF-κB activation. In TPC-1 cells overexpressing LDOC1, the nuclear translocation of p65 in response to rTGF-β1 treatment was decreased as compared with that in Lv-NC cells (Fig. 6a); furthermore, the expression level of IκBα in LDOC1-overexpressing was higher than that in control cells. Thus, we next determined whether LDOC1-mediated ablation of the NF-κB survival signal was sufficient for affecting the cell cycle distribution of TPC-1 cells under exogenous TGF-β1 stimulation. As per our expectation, the results of flow cytometry analysis showed that the DNA profile exhibited a statistically significant shift to a subG1 position in Lv-LDOC1 cells as compared with that in Lv-NC (Fig. 6b).

Lastly, we performed CCK-8 assays and evaluated whether LDOC1 overexpression influences the viability of TPC-1 cells under TGF-β1 stimulation. As compared with Lv-NC cells, Lv-LDOC1 cells exhibited drastically lower cell viability after treatment for 12 h with rTGF-β1 (5 ng/mL) (Fig. 6c); this indicated that LDOC1 overexpression, which leads to the inhibition of NF-κB activation, might partially restore TGF-β1 signal transduction. Furthermore, these results confirmed that the antiproliferative effect of TGF-β1 is mediated by the NF-κB survival pathway (Fig. 6b, c). Collectively, the results of our analyses suggest that LDOC1-mediated NF-κB repression plays a crucial role in the restoration of PTC cell responsiveness to TGF-β1 antiproliferative signaling.

## Discussion

The involvement of aberrant deregulation of oncogene expression in human PTC has been well documented. However, little is known about the role in PTC of tumor suppressors implicated in cellular processes associated with tumorigenesis or tumor progression. Furthermore, certain PTCs have been reported to not exhibit alterations in commonly implicated oncogenes. Therefore, genes and mechanisms that currently remain unidentified might also play critical roles in thyroid carcinogenesis.

*LDOC1* was previously identified as a differentially expressed gene in certain human cancers; for example, the gene was differentially expressed in pancreatic and gastric cancer cell lines [16]. In this study, we used a large number of specimens to compare the expression of LDOC1 in PTC tissues and normal samples. Our results demonstrated that *LDOC1* mRNA expression was downregulated in PTC specimens. Furthermore, our functional studies supported the hypothesis that LDOC1 expression is associated with antiproliferative and apoptotic effects in thyroid cells.

Here, we also observed that the reduction in *LDOC1* mRNA levels was associated with an increase in the size of primary PTC tumors, and that *LDOC1* mRNA was almost undetectable in certain specimens in which the tumor size was >4 cm. Notably, in certain PTC samples in which LDOC1 was detectable, the protein was delocalized from the nucleus to the cytoplasm; by contrast, LDOC1 localized predominantly to the nucleus in normal tissues. The protein sequence of LDOC1 suggests that LDOC1 might interact with other transcription factors through its leucine zipper domain or regulate cell signaling by providing an SH3-binding surface for proteins that function in signaling cascades. The nucleus-to-cytoplasm shift in PTC might interfere with the regular function of LDOC1 [18].

As expected, restoration of *LDOC1* expression in a PTC cell line characterized by very low levels of LDOC1 expression was associated with a proapoptotic effect and a reduction in cell proliferation. Cell numbers were drastically decreased in TPC-1 cells in which the LDOC1 cDNA was expressed by means of lentiviral infection, and these cells also exhibited a shift of the DNA profile to a subG1 position, as revealed by flow cytometric analysis of cell cycle progression. Furthermore, Annexin V-APC/7-AAD staining also confirmed an increase in the level of apoptosis of LDOC1-overexpressing cells. Together, the data reported herein are consistent with the view that LDOC1 downregulation is involved in the process of PTC tumorigenesis and/or progression. The results of this study also suggest that the mechanism by which *LDOC1* affects cell growth and apoptosis involves LDOC1 regulation of NF-κB survival signaling, particularly in response to various extracellular stimuli such as TNFα and TGF-β1. Thus, LDOC1 might serve as a promising novel biomarker and therapeutic target for the diagnosis and treatment of PTC and other cancers.

NF-κB is a widely recognized transcription factor that regulates the expression of numerous genes involved in inflammation, immune response, aberrant cell growth, and apoptosis [37]. High constitutive expression of NF-κB is a primary feature of cancer cells but not normal cells, and this indicates the crucial role of NF-κB in regulating tumorigenesis [38–40]. In normal cells, NF-κB localizes in the cytoplasm by binding to its inhibitor IκBα. After cells are exposed to various stimuli, the IκBα protein is phosphorylated, ubiquitinated, and degraded, following which the released NF-κB complexes translocate to the nucleus and regulate the transcription of target genes [41]. TNFα is commonly recognized as a potent inducer of NF-κB activation in several cell types. NF-κB has been shown to associate with C/EBP transcription factors through an interaction between the NF-κB Rel homology domain and a leucine zipper-like motif in C/EBP family members. The LDOC1 protein also possesses a basic region-leucine zipper and is localized in the nucleus, and thus LDOC1 might also directly interact with NF-κB, although the detailed mechanism involved remains unclear. In this study, we confirmed that (a) LDOC1 expression was downregulated and was correlated with nuclear P65 content in PTC tissues; (b) LDOC1 overexpression exerted antiproliferative and proapoptotic effects; and (c) LDOC1 inhibited both basal and TNFα-induced NF-κB activation. Collectively, these data support the notion that a loss of LDOC1 expression might enhance both the constitutive and the inducible activation of NF-κB signaling, and, consequently, contribute to the process of thyroid carcinogenesis.

The TGF-β1 pathway plays a key role in the control of thyroid cell numbers by mediating cell growth and apoptotic effects under normal physiological conditions. However, TGF-β1 also acts as a tumor promoter during epithelial-mesenchymal transdifferentiation and in mesenchyme-derived tumors. Therefore, rTGF-β1 plays a dual role in the regulation of epithelial cells depending on the type and the state of the cells, potentially inducing either apoptosis or survival. Although the mechanisms by which thyroid tumor cells bypass TGF-β1-mediated inhibitory signaling are not fully understood, the effect of TGF-β1 on thyrocyte survival has been shown to be mediated by TGF-β1 activation of NF-κB [36]. The data reported herein demonstrated that the upregulation of LDOC1 expression in a PTC cell line, TPC-1, resulted in reductions of NF-κB activation and cell viability in response to rTGF-β1 stimulation; this indicates that the inhibition of LDOC1 expression might represent a critical step in PTC cells, a step that would lead to an increase in NF-κB activity and thereby impair TGF-β1 inhibitory signaling. Thus, this study provides a potential connection between TGF-β1 effects and NF-κB survival signaling in human thyroid carcinogenesis. Our finding might have substantial practical clinical implications, because therapies based on targeting NF-κB are currently of considerable interest. Nevertheless, multiple parallel signal transduction pathways might also contribute to the disruption of TGF-β1 inhibitory signaling in TPC-1 cells; for example, TGF-β1 signal transduction can also be mediated by a non-Smad pathway [42].

## Conclusions

In summary, this study highlights the functionality of LDOC1 in thyroid carcinogenesis. We demonstrated that LDOC1 is downregulated in PTC tumor tissues and in thyroid cancer cell lines (BCPAP and TPC-1), and that LDOC1 expresion is relevant to tumor size and nuclear P65 content in PTC tissues. Furthermore, we found that LDOC1 expression sensitizes thyroid cancer cells to apoptosis by suppressing both basal and TNFα-induced activation of NF-κB signaling, and, consequently, increases the responsiveness of these cells to TGF-β1 inhibitory signaling. Therefore, LDOC1 targeting might yield substantial diagnostic and therapeutic benefits for patients with PTC.

## Abbreviations

FBS: fetal bovine serum
HRP: horseradish peroxidase
Lv-LDOC1: lentiviral vector harboring the *LDOC1* cDNA
Lv-NC: empty vector (negative control)
MOI: multiplicity of infection
PARP: poly ADP-ribose polymerase
PTC: papillary thyroid carcinoma
qRT-PCR: quantitative reverse transcription-PCR
UNT: untransduced
